# Universal use of face mask for the prevention of the spread of COVID-19 in community settings in a South-western State of Nigeria: willingness and barriers

**DOI:** 10.1186/s13756-023-01267-3

**Published:** 2023-07-05

**Authors:** Folasade T. Ogunsola, Chioma S. Ejekam, Mobolanle Balogun, Igwilo Ugonna, Oluwakemi Odukoya, Oyinlola Oduyebo, Wasiu L. Adeyemo, Rita O. Oladele, Sulaimon A. Akanmu

**Affiliations:** 1 Department Medical Microbiology, College of Medicine, University of Lagos, Idiaraba, Lagos, Nigeria; 2 Department of Community Health and Primary Care, College of Medicine, University of Lagos, Idiaraba, Lagos, Nigeria; 3 Department of Oral and Maxillofacial Surgery, College of Medicine, University of Lagos, Idiaraba, Lagos, Nigeria; 4 Department of Haematology and Blood Transfusion, College of Medicine, University of Lagos, Idiaraba, Lagos, Nigeria

**Keywords:** Willingness to wear facemask, Barriers to wearing face mask, Compliance to face mask use, Universal use of mask, Community mask use, SARS-CoV-2, Face mask use for COVID-19, Lagos Nigeria

## Abstract

**Background and objectives:**

One of the major drivers of the novel coronavirus (SARS‐CoV‐2) pandemic is community transmission. Nigeria, like other countries globally, took to strict preventive public health measures including good respiratory and hand hygiene, physical distancing, and the use of face mask to control the spread of COVID-19 disease. Furthermore, the government of Lagos State in Nigeria made a pronouncement on the universal use of face mask in the community. While the use of face masks has proven to be an effective barrier to the transmission of respiratory diseases, its use in the community is uncommon. This study assessed the willingness and compliance with wearing face masks for the reduction of the community spread of COVID-19 and identified possible barriers to use of mask among residents in Lagos State.

**Methods:**

This was a descriptive cross-sectional study, that surveyed 552 respondents who were adult residents of Lagos State. Data collection was quantitative, using a pretested, interviewer-administered questionnaire, and findings were presented in frequencies and percentages. Pearson’s chi-square and logistic regression analyses were used to test the association between variables. The level of significance was set at 5%.

**Results:**

A majority (75.7%) of the respondents were willing to wear a face mask in public areas but only 21.9% of the respondents were willing to wear a mask at all times. The most identified barriers to wearing mask were discomfort (72.5%) and inconvenience (77.7%). Two-thirds of the respondents reported they were compliant with always wearing a face mask when leaving home. Only 15.0% of the respondents wore the mask continuously and appropriately, covering the nose and mouth. Having a post-secondary education and being older (40 years and above) were found to be positive predictors of both willingness to wear a mask and compliance with universal mask policy (wearing masks continuously and appropriately).

**Conclusion:**

Our findings suggest that willingness to wear a face mask influences compliance, and that having a post-secondary education and being older (> 40 years) were positive predictors of both willingness to wear a mask and compliance with universal mask policy (wearing it continuously and correctly). The major barriers to wearing masks were discomfort and inconvenience. Effective risk communication strategies to reach diverse groups for better compliance with public health measures are urgently needed even for the future.

## Introduction

The novel coronavirus (SARS‐CoV‐2) that causes coronavirus disease 2019 (COVID‐19) occurred in Wuhan, China leading to a pandemic [1]. In Nigeria, the earlier cases were imported but subsequently, the driver of the outbreak was largely continued community transmission [1]. To prevent and control infectious respiratory diseases like the novel coronavirus, Nigeria has followed the first line of defence for preventing exposures by using control measures, such as isolation, quarantine, and restricting or closing group gatherings [1–4]. There was also massive public education on measures such as good respiratory hygiene/cough etiquette, regular hand washing, and use of alcohol-based hand rub [5].

The use of personal respiratory protection has been said to provide the last line of defence in the hierarchy of safety and health controls [6]. Prevention with the use of personal respiratory protection uses two methods: (1) those meant to prevent inhalation by the user (i.e., respirator) and (2) those meant to protect persons around the user by limiting exhaled particles (such as mask) [4]. Following the evidence that COVID-19 could be transmitted before symptom onset, community transmission might be reduced if everyone including people who have been infected but are asymptomatic and contagious, wear face masks [7]. There was a global shortfall of surgical masks (these are quality medical masks designed with 3-layer non-woven fabric to block large-particle droplets or splashes through cough, sneezing, and talking), but anecdotal evidence has shown that handmade cotton masks were protective even among healthcare workers during previous epidemics and pandemics [8–11]. A simple, locally made, washable mask may be a solution if commercial masks are unavailable [9–11].

Studies have documented poor compliance with the use of face masks in previous pandemics and have identified barriers like low perceived susceptibility to the disease, lack of knowledge about perceived benefits, barrier to social interaction, appearance and fashion trends, anxiety about face covering and comfort, etc. [12, 13]. The use of face masks has proven to be an effective barrier to reducing the transmission of respiratory diseases. However, its use in the community is uncommon. To reduce the community spread of SARS-CoV-2 in the early phase of the pandemic, the government of Lagos State made a pronouncement on universal community face masking. This study aimed to assess the willingness of residents and barriers in Lagos State towards wearing face masks for the reduction of community spread of SARS-CoV-2. The objectives of this study were to assess what respondents knew about COVID-19 transmission and public health preventive measures, to assess respondents’ willingness to wear face masks for the prevention of COVID-19, to identify the barriers to wearing face masks for the prevention of COVID-19, and to assess compliance with the universal face mask policy in the public area**.**

## Methods

### Background to the study site

Lagos State was created on May 27, 1967, and took off as an administrative entity on April 11, 1968. It is the smallest state in Nigeria occupying 0.39% of the total land and harbouring about 10% of the nation’s population with a population density of 5,926 persons per sq.km [14–16] Lagos State is divided into five administrative divisions namely Ikeja, Badagry, Ikorodu, Lagos, and Epe. These divisions have variable numbers of Local Government Areas (LGAs): Ikeja division (8 LGAs), Badagry division (4 LGAs), Ikorodu division (1 LGA), Lagos division (5 LGAs), and Epe division (2 LGAs). Epe and Badagry divisions are designated as rural divisions while, Ikeja, Lagos, and Ikorodu divisions are urban divisions. In total, there are twenty LGAs in Lagos State.

### Study design and population

We conducted a cross-sectional study among adult residents of Lagos State. We included residents who were 18 years and above and had been living in Lagos State for at least 6 months. Visitors and persons with a health condition that made mask-wearing unacceptable (for example, severe respiratory diseases) were excluded from participation.

### Sample size determination

The sample size was determined for the study using Cochran’s formula [17], assuming standard normal deviate at 95% (1.96), accepted error margin of 5%, and *p* (proportion of respondents willing to wear face masks from a previous study) of 58% [18]. We calculated a minimum sample size of 375 and increased the sample size to 552 to account for design effect. The assessment of the reliability of the tool gave a Cronbach’s alpha of 0.89.

### Sampling technique

A multistage sampling technique was used to select respondents from the five administrative divisions in Lagos State. First, five LGAs were selected from the 20 LGAs in Lagos State using simple random sampling and the sample size was equally allocated to each. The selected LGAs were Alimosho, Kosofe, Lagos Island, Oshodi, and Surulere. Second, two wards were selected from each LGA by simple random sampling making a total of 10 wards. Third, five streets were selected from each ward by simple random sampling making a total of 50 streets. Fourth, 10 houses were selected from each street by systematic random sampling. Fifth, one household per house was selected using simple random sampling. Lastly, one eligible respondent per household was selected. Where there was more than one eligible respondent, simple random sampling was used to select one respondent in the household.

### Data collection technique and procedure

Data was obtained quantitatively with a structured, interviewer-administered questionnaire and entered electronically using the KoBoToolbox app (Harvard Humanitarian Initiative, Cambridge, Massachusetts, USA). Data collection was between August to October, 2020. A semi-structured questionnaire was developed from the review of available published literature. The questionnaire had four Sections; A to E. Section A obtained information on the respondents’ sociodemographic characteristics; B assessed general knowledge of COVID-19 and transmission and asked questions about the cause of COVID-19 disease, which system of the body most affected, common symptoms, recommended number of days for isolation, availability of an effective cure for COVID-19, the transmission of COVID-19, existing preventive measures, materials that could serve as masks and proper use of mask; C assessed willingness to wear a mask for prevention of COVID-19 in the scenarios; D assessed the barriers to wearing a mask for the prevention of COVID-19 and section E assessed the compliance to other COVID-19 public health preventive measures.

The questionnaire was pre-tested amongst 20 respondents in another LGA not included in the study. The data obtained was analysed and used to modify the questionnaire for wording, content, and coding of responses.

Five research assistants with a minimum of Ordinary National Diploma (OND) who are primarily health workers (involved in outreach and field works) and speak the local languages were trained to assist with the administration of the questionnaires. They adhered to COVID-19 preventive public health measures such as physical distancing and proper use of face masks while on the field and use of alcohol-based hand rub.

### Data analysis

Data entry, cleaning, and analysis were done using IBM Statistical Package for Social Sciences (SPSS) version 21. Data was presented in frequency tables. Categorical variables were summarized using simple proportions while Student’s t-test and chi-square were used to test for association between variables. The outcome variables were willingness to wear a mask and compliance with the universal mask policy (correct use of face mask when leaving home for public spaces) while the explanatory variables were age, sex, the highest level of education, and employment status. Logistic regression analysis was used to examine multivariate associations between respondent characteristics and willingness to wear masks as well as compliance with the universal face mask policy. Variables with *p* values < 0.05 in the bivariate analysis were included in the logistic regression models. Level of significance was set at 0.05.

## Results

Overall, 552 respondents participated in the study. The mean age was 38.6 years + 17.4 standard deviation. About half (49.5%) of the respondents completed the secondary level of education while 81.2% (448/552) of the respondents were employed. (Table 1).

**Table 1:** Socio-demographic variables

Variable	Frequency (N = 552)	Percentage (%)
*Age*		
< 40 years	328	59.4
≥ 40 years	224	40.6
Mean age	38.60 ± 17.40	
*Sex*		
Male	256	46.4
Female	296	53.6
*Highest level of education*		
No formal education	8	1.4
Primary school uncompleted	6	1.1
Primary school completed	48	8.7
Secondary school uncompleted	34	6.2
Secondary school completed	273	49.5
Religious schooling only	2	0.4
Literacy classes only	3	0.5
Post-secondary	178	32.2
*Employment status*		
Employed	448	81.2
Unemployed	104	18.8
*Occupational status (for employed)*	(n = 448)	
Senior professional	31	5.62
Intermediate professional	66	11.9
Junior professional/skilled	100	18.1
Semi-skilled	211	38.2
Unskilled	37	6.7
Others	3	19.4
*Status (for unemployed)*	(n = 104)	
Housewife	25	4.5
Student	41	7.4
Apprentice	13	2.4
Retiree	13	11.1
Others	12	2.2

Table 2 highlights the knowledge of COVID-19 transmission among the respondents. The majority of the respondents knew that COVID-19 was a virus 90.9% (502/552) and that it affected the respiratory tract 81.5% (450/552). High proportions of the respondents identified common symptoms of COVID-19 as; Fever 78.1% (431/552), Cough 94.1% (519/552), Difficulty breathing 83.3% (460/552), and Runny nose/catarrh 59.1% (326/552). High proportions of the respondents knew about preventive measures for COVID-19 as washing hands with soap 97.3% (537/552), use of hand sanitizers 88.4% (488/552), staying two metres apart from anyone 74.1% (409/552), and wearing a face mask outside the home 85.5%.

**Table 2:** Knowledge of COVID-19 transmission

Variable	Frequency (n = 552)	Percentage (%)
*COVID-19 is caused by a virus*		
Yes	502	90.9
No	9	1.6
Uncertain	41	7.4
*COVID-19 affects the respiratory tract*		
Yes	450	81.5
No	5	0.9
Uncertain	97	17.6
Mode of transmission of COVID-19*		
Close contact with an infected person with symptoms	430	77.9
Close contact with an infected person who has no symptoms	204	37.0
Contact with infected surfaces or objects	217	39.3
Breathing infected air	242	43.8
Unwashed hands	278	50.4
Other	9	1.6
Common symptoms of COVID-19*		
Fever	431	78.1
Cough	519	94.1
Chills	230	41.7
Tiredness	221	40.4
Difficulty in breathing	460	83.3
Sore throat	270	48.9
Runny nose/catarrh	326	59.1
Recent loss of sense of taste or smell	151	27.4
*Number of days for COVID-19 self-isolation following exposure is 14 days*		
Yes	347	62.9
No	205	37.1
*Presence of an effective cure for COVID-19*		
Yes	61	11.1
No	347	62.9
Uncertain	144	26.1
Places where possible COVID-19 transmission can occur*		
Markets/malls	276	50.0
Schools/workplaces	253	45.8
Motor parks/public transportation	343	62.1
Churches/Mosques	376	68.1
Parties/mass gatherings	404	73.2
Anywhere	324	58.7
Preventive measures known about COVID-19*		
Wash hands with soap and water	537	97.3
Use alcohol hand rub/sanitizer	488	88.4
Cleaning surfaces regularly	268	48.6
Cough into your elbow or tissue paper and dispose immediately	261	47.3
Avoid touching your face, nose, mouth, and eyes	240	43.5
Self-isolate if you feel sick	237	42.9
Avoid public gatherings and public places	429	66.1
Stay at least 2 m apart from anyone	409	74.1
Wear a mask when outside your home	472	85.5

*Multiple responses

About 56.9% (314/552) were willing to wear face masks and only 20.7% were extremely willing to wear face masks as an added preventive measure against COVID-19 while, just over a third of the respondents (39.3%-217/552)) wore mask most of the time, each time they were outside the home (Table 3). Table 4 highlights the barriers to wearing masks for the prevention of COVID-19. A higher proportion of the respondents noted that wearing any kind of mask was discomforting 72.5%(400/552) and that it was also an inconvenience 77.7%.

**Table 3:** Willingness to wear facemasks for the prevention of COVID-19

Variable	Frequency (n = 552)	Percentage (%)
*Willing to wear a face mask as an added effective preventive measure against COVID-19*		
Not willing at all	11	1.9
Unwilling	23	4.1
Somewhat willing	90	16.3
Willing	314	56.9
Extremely willing	114	20.7
*Willing to wear a face mask each time outside the home*		
None of the time	21	3.8
Some of the time	194	35.1
Most of the time	217	39.3
All of the time	121	21.9
*Willing to wear a face mask in public areas such as malls, parks, markets, churches, mosques*		
Not willing at all	12	2.1
Unwilling	13	2.4
Somewhat willing	109	19.8
Willing	309	55.9
Extremely willing	109	19.8
*Willing to wear face masks if family and friends also wear one*		
Not willing at all	13	2.4
Unwilling	29	5.3
Somewhat willing	97	17.6
Willing	335	60.9
Extremely willing	78	14.1
*Willing to wear a facemask if health authority regulation requires everyone to wear one*		
Not willing at all	3	0.5
Unwilling	12	2.1
Somewhat willing	63	11.4
Willing	357	64.7
Extremely willing	117	21.2
*Willing to wear a face mask if a media campaign asks everyone to do so*		
Not willing at all	3	0.5
Unwilling	22	3.9
Somewhat willing	98	17.8
Willing	350	63.4
Extremely willing	79	14.3
*Willing to wear a face mask if healthcare professional advises doing so*		
Not willing at all	1	0.2
Unwilling	4	0.7
Somewhat willing	48	8.7
Willing	364	65.9
Extremely willing	135	24.5
*Willing to wear a face mask if work/school policy requires it*		
Unwilling	4	0.7
Somewhat willing	35	6.3
Willing	353	63.9
Extremely willing	160	28.9

**Table 4:** Barriers to wearing a mask for the prevention of Covid 19

Variable	Frequency (n = 552)	Percentage (%)
*Cannot afford surgical/medical grade face masks*		
Yes	195	35.3
No	357	64.7
*Cannot find medical-grade face mask even though it is affordable*		
Yes	156	28.3
No	396	71.7
*Wearing any other face mask apart from the medical grade mask will not offer any protection*		
Yes	64	11.6
No	488	88.4
*Do not know how to make a cloth mask or improvise one*		
Yes	244	44.2
No	308	55.8
*Wearing any kind of mask causes discomfort*		
Yes	400	72.5
No	152	27.5
*Forget to wear mask on leaving home*		
Yes	189	34.2
No	363	65.8
*Mask not the right fit for face (small fit/too big)*		
Yes	239	43.3
No	313	56.7
*Cannot wear face mask due to the hot weather*		
Yes	185	33.5
No	367	66.5
*Inconveniencing to wear face mask*		
Yes	429	77.7
No	123	22.3
*Embarrassing to wear face mask*		
Yes	89	16.1
No	463	83.9
*People make me feel contagious and stigmatize me*		
Yes	86	15.6
No	466	84.4
*Family and friends will not support me to wear a face mask*		
Yes	27	4.9
No	525	95.1
*It is unattractive to wear a face mask*		
Yes	241	43.7
No	311	56.3
*Do not know how to appropriately don and doff a mask*		
Yes	138	25.0
No	414	75.0

Regarding the level of compliance of the respondents with the universal facemask policy, 32.4%(179/552) of the respondents noted that they always wore a face mask when stepping out of the house into public spaces. Only 14.9%(82/552) of the respondents noted that they always put their masks fully on(covering nose and mouth) and only 3.1%(17/552) of the respondents have always seen others wear mask when in enclosed public spaces (Table 5).

**Table 5:** Compliance with universal facemask policy

Variable	Frequency (n = 552)	Percentage (%)
*Aware of Lagos State policy of universal use of face mask when leaving home or going to public places*		
Yes	551	99.8
No	1	0.2
*Always wear a face mask when leaving home*		
Yes	331	59.9
No	221	40.0
*How often did you wear face mask when stepping out of the house in the past 1 week*		
Never	32	5.8
Rarely	147	26.6
Quite Often	194	35.1
Always	179	32.4
*When wearing the mask in public places, how often is it kept fully on (Covering nose and mouth)*		
Never	22	3.9
Rarely	98	17.8
Sometimes	152	27.5
Quite Often	198	35.9
Always	82	14.9
*How often you have seen others wear mask whenever they were in enclosed public spaces in the past 1 week*		
Never	7	1.3
Rarely	174	31.5
Sometimes	204	36.9
Quite Often	150	27.2
Always	17	3.1

In bivariate analyses, having no formal education was significantly associated with unwillingness to wear a facemask as an added preventive measure (*p* < 0.001) [Table 6]. Being older (aged > 40 years) and having a post-secondary level of education were significantly associated with always wearing face masks correctly when leaving home for public (*p* < 0.002 and < 0.001) respectively (Table 7).

**Table 6:** Association between socio-demographic variables and willingness to wear a face mask as an added effective preventive measure

Variable	Willingness to wear a face mask as an added effective preventive measure Frequency (%)			Χ^2^	*p* value
	Unwilling	Somewhat willing	Willingly		
*Age*					
< 40 years	24 (7.3)	61 (18.6)	243 (74.1)	5.607	0.059
≥ 40 years	10 (4.5)	29 (12.9)	185 (82.6)		
*Sex*					
Male	18 (7.0)	34 (13.3)	204 (79.7)	3.550	0.168
Female	16 (5.4)	56 (18.9)	224 (75.7)		
*Highest level of education*					
No formal education	4 (50.0)	1 (12.5)	3 (37.5)	50.602	< 0.001
Primary school educated	5 (9.3)	12 (22.2)	37 (68.5)		
Secondary school educated	21 (6.9)	59 (19.2)	227 (73.9)		
Informally educated	1 (20.0)	2 (40.0)	2 (40.0)		
Post-secondary school educated	3 (1.7)	16 (9.0)	159 (89.4)		
*Employment status*					
Employed	26 (5.8)	73 (16.3)	349 (77.9)	0.5299	0.713
Unemployed	8 (7.7)	17 (16.3)	79 (76.1)

**Table 7:** Association between socio-demographic variables and frequency of correct use of face mask when leaving home for public spaces

Variable	Frequency of correct and continuous use of face mask when in public spaces (compliance to universal mask policy) (%)					Χ^2^	*p* value
	Always	Quite often	Sometimes	Rarely	Never		
*Age*							
< 40 years	32 (9.8)	123 (37.5)	95 (29.0)	62 (18.9)	16 (4.9)	17.560	0.002
≥ 40 years	50 (22.3)	75 (33.5)	57 (25.4)	36 (16.1)	6 (2.7)		
*Sex*							
Male	47 (18.4)	93 (36.3)	70 (27.3)	37 (14.5)	9 (3.5)	7.175	0.127
Female	35 (11.8)	105 (35.5)	82 (27.7)	61 (20.6)	13 (4.4)		
*Highest level of education*							
No formal education	1 (12.5)	1 (12.5)	1 (12.5)	2 (25.0)	3 (37.5)	76.505	< 0.001
Primary school educated	5 (9.3)	12 (22.2)	16 (29.6)	17 (31.5)	4 (7.4)		
Secondary school educated	38 (12.4)	98 (31.9)	92 (30.0)	67 (21.8)	12 (3.9)		
Informally educated	0 (0.0)	2 (40.0)	2 (40.0)	0 (0.0)	1 (20.0)		
Post-secondary school educated	38 (21.3)	85 (47.8)	41 (23.0)	12 (6.7)	2 (1.1)		
*Employment status*							
Employed	66 (14.7)	165 (36.8)	123 (27.5)	74 (16.5)	20 (4.5)	4.055	0.399
Unemployed	16 (15.4)	33 (31.7)	29 (27.9)	24 (23.1)	2 (1.9)		

A multivariate analysis was done to control for the significant explanatory variables from the bivariate analysis of the main outcome variables—willingness to wear mask and compliance to universal mask policy. The outputs are presented in Tables 8 and 9.

**Table 8:** Predictors of willingness to wear mask

Variable	OR	95% CI OR		*p* value	AOR	95% CI AOR		*p* value
		Lower	Upper			Lower	Upper	
*Age category (years)*								
< 40 years	Ref.				Ref.			
≥ 40 years	1.68	1.1.0	2.56	0.016*	1.83	1.16	2.90	0.010*
*Gender*								
Male	Ref.							
Female	0.82	0.55	1.23	0.34				
*Highest level of education*								
No formal education	Ref.				Ref.			
Primary education	6.72	1.46	31.01	0.015*	6.34	1.39	28.98	0.017*
Secondary education	8.83	2.09	37.37	0.003*	10.34	2.44	43.73	0.002*
Informal education	2.24	0.27	19.01	0.46	2.42	0.42	20.43	0.417
Tertiary education	26.24	5.85	117.63	< 0.001*	29.02	6.49	129.74	< 0.001*
*Employment status*								
Unemployed	Ref.							
Employment	1.13	0.69	1.87	0.62				

*statistically significant

**Table 9:** Predictors of compliance with universal mask policy

Variable	OR	95% CI OR		*p* value	AOR	95% CI AOR		*p* value
		Lower	Upper			Lower	Upper	
*Age category (years)*								
< 40 years	Ref.				Ref.			
≥ 40 years	1.63	1.21	2.57	0.002*	1.80	1.29	2.52	0.001*
*Gender*								
Male	Ref.				Ref.			
Female	0.69	0.51	0.93	0.016*	0.91	0.66	1.24	0.538
*Highest level of education*								
No formal education	Ref.				Ref.			
Primary education	3.82	0.81	18.27	0.094	3.62	0.79	16.61	0.098
Secondary education	6.85	1.52	30.89	0.012*	8.36	1.92	36.43	0.005*
Informal education	5.11	0.59	44.44	0.139	5.86	0.71	48.46	0.101
Tertiary education	17.87	3.91	81.87	< 0.001*	19.91	4.50	88.04	< 0.001*
*Employment status*								
Unemployed	Ref.							
Employment	1.13	0.77	1.66	0.53				

*statistically significant

The logistic regression analysis indicated that two out of the four explanatory variable including age category (years) and highest level of education predicted willingness to wear mask. (Table 8).

Fitting for factors with *p* values less than 0.05 from the univariate logistic regression into multivariate logistic regression, only age category (years) and highest level of education were included in the final regression model to predict willingness to wear mask. (Table 8).

Holding other variables constant, the odds of wearing mask was 1.8 times more, among 40 years or older people compared to those less than 40 years (AOR = 1.82, 95% Cl 1.16–2.90, *p* = 0.09). Also, increase in educational level was significantly associated with willingness to wear mask thus; holding other factors constant, individuals with tertiary education were 28.8 times more likely to wear mask than those with no formal education, those with secondary and primary education were 10.3 and 6.3 times more likely to wear mask than those with no formal education [tertiary (AOR = 26.24, 95% Cl 6.47–127.81, *p* < 0.001), secondary (AOR = 10.3, 95% Cl 2.44–43.74, *p* = 0.001) and primary (AOR = 6.3, 95% Cl 1.39–28.91, *p* = 0.017)] respectively as presented in Table 8.

On compliance with mask universal mask policy, three out the four factors including age category (years), gender and highest level of education predicted compliance with universal mask policy in the univariate logistic regression model. (Table 9) In the final multivariate logistic regression, we adjusted for only the three predictor variables significantly associated with compliance with universal mask policy. Individuals in the age category ≥ 40 years were 1.3 times more likely to comply with the universal mask policy (AOR = 1.78, Cl 1.28–2.47, *p* = 0.001) compared to those in 40 years category. Increase in educational level was a significant predictor of universal compliance with mask policy thus; holding other factors constant, individuals with tertiary education were 20.1 times more likely to comply with the universal mask policy than those without formal education, those who attained secondary level education were 8.5 times more likely to comply with the universal mask policy than those without formal education [tertiary (AOR = 20.1, 95% Cl 4.54–88.97, *p* < 0.001), secondary (AOR = 8.53, 95% Cl 1.96–37.15, *p* = 0.004)] respectively. However, gender was not a significant predictor of the universal mask policy in this model. (Table 9).

## Discussion

This study was carried out among 552 respondents across Lagos State (a South-western State) in Nigeria to assess the willingness and barriers of Lagos State residents to wearing face mask for the reduction of the community spread of SARS-CoV-2. A significant proportion of our respondents were less than 40 years of age while the majority of our respondents were employed. This study revealed that our respondents have basic knowledge about SARS-CoV-2 as a virus that causes COVID-19, which primarily infects the respiratory system including its modes of transmission. This was expected because of the level of awareness already attained in the community about the COVID-19 pandemic [1–4]. This is similar to findings from previous studies [1–4]; and this knowledge was relatively high in populations where awareness had been raised [19–21]. It is surprising though to have observed that despite the massive awareness campaigns regarding SARS-CoV-2 transmission from asymptomatic persons, that very few of the respondents identified close contact with an infected person who has no symptoms and contact with infected surfaces or objects as possible mode of COVID-19 transmission. This poor knowledge that asymptomatic carriers can transmit the virus has also been reported in another study in Nigeria [20]. This is an important finding because contact with infected surfaces or objects necessitates proper hand hygiene as the hands are a major means of germ transmission.

Similar to findings from previous studies [22–24], our study showed that majority of the respondents identified cough, fever, and difficulty in breathing as common symptoms of COVID-19. Interestingly, very few of the respondents in the previous studies were aware of the recent loss of sense of taste or smell, tiredness, sore throat as also possible symptoms of COVID-19 despite observing very high knowledge of the virus affecting the respiratory tracts. The study found that just about a third of the respondents knew the recommended number of days (14 days) of isolation for suspected exposure to the SARS-CoV-2 virus and that there was currently no effective cure for COVID-19 disease. This is contrary to a previous study done in Nigeria which reported that the majority (over 90%) of their respondents were aware that there is currently no cure for the COVID-19 disease [20]. This difference is likely because their study population was educated Nigerians with access to the internet and computers [20], while our respondents were randomly selected from the local communities.

On assessing the knowledge of public health preventive measures, our study found that the majority of the respondents were aware of the public health preventive measures against COVID-19: wash hands with soap and water, use alcohol hand rub/sanitizer, wear a mask when outside your home, stay at least 2 m apart from anyone, and avoid public gatherings and public places. However, there was poor knowledge of other preventive measures like coughing into your elbow or tissue paper and dispose of immediately, cleaning surfaces regularly, avoiding touching your face, nose, mouth, and eyes, and self-isolation if one feels sick. Web-based surveys in the educated population have reported very high knowledge of these preventive measures contrary to our findings [20, 25]. It is therefore crucial to mobilize trusted members of the community to develop and drive innovative community-tailored awareness strategies. This will include dialogue, and communication tools in the local language suited for individuals at different spectrums of education.

Lagos State government in Nigeria was the first government to pass a ‘mask-up’ Lagos policy with existing penalties for defaulters. Our study found that a high proportion of the respondents were willing to wear a face mask as an added effective preventive measure against COVID-19, wear mask in public areas, and if health regulations required everyone to wear one including media campaigns and healthcare professionals advice. However, only a few proportions indicated a willingness to wear a facemask at all times outside the home. This could lead to the further spread of the SARS-CoV-2 virus and a subsequent rise in the number of cases state-wide leading to further depletion of the finite resources used by the state in combating the pandemic. This finding was expected given the level of behaviour change communication and educational campaigns about the effectiveness of community face-masking to prevent COVID-19 and the alternative of making and using readily available cloth masks [26]. Our study found that increase in educational level was a positive predictor of willingness to wear a face mask. The respondents who had tertiary education were most willing to wear mask. Hence information, education, and communication concerning COVID-19 public health preventive measures including wearing face mask must be carefully tailored to suit diverse populations especially those with no formal education. This is needed to bridge behaviour change communication gaps. Age was found to be a significant predictor of willingness to wear mask. The older population (≥ 40 years old) were more willing to wear a face mask as an added preventive measure against COVID-19. This would be an advantage since a higher risk of severe forms of COVID-19 occurs in the older age groups and those with co-morbidities [27]. Furthermore, this may imply that the perceived risk was higher among the older age group. It is therefore imperative to target appropriate behaviour change communication interventions and risk communications strategies at the younger age group who incidently are the majority in Nigeria population. In addition, lack of adoption of the COVID-19 precautionary measures including wearing face mask in the younger age group could cause increased incidence and persistence of the community transmission of the COVID-19 virus and other respiratory viruses.

Regarding the barriers to wearing mask for the prevention of COVID-19, the major barriers identified among the respondents in our study were that face mask causes discomfort and it was not convenient wearing one. It was more interesting to note that inability to afford a medical-grade mask was not a barrier to using a mask in the majority of the respondents. It may indicate that the community was aware of the various options for less costly face-covering hence the cost of masking up was not a barrier. Similar to the findings from our study, a community study done in Saudi Arabia reported discomfort and inconvenience among others as major barriers to wearing face mask for the prevention of COVID-19 [28].

Almost all the respondents in the current study reported being aware of Lagos State policy concerning the universal use of face mask when leaving home or going to public places yet the findings revealed that not everyone complied with this directive when leaving home. In our study, just over half reported wearing a face mask when leaving home. Compliance with proper and consistent use of mask has remained a challenge for individuals despite the massive advocacy and understanding of the importance of using mask through diverse media communication and engagement channels. A possible reason for the high awareness of the mask-wearing policy but low compliance may be similar to that identified in the aforementioned Saudi-Arabian study were the respondents reported inconvenience. Despite having the cloth mask, the quality of the mask such as breathability and cut of the mask is crucial for comfort, especially for the very humid weather of the state and social patterns of the individuals in the State. It was often a common site to see various unsuitable sizes and cloth textures being used to mask up.

To reduce the community spread of COVID-19, high compliance with the use is of great importance and wider benefit when practiced together with other measures like social distancing [28]. In assessing compliance of the respondents within a 1-week recall period, our study found that just a third wore face mask always when stepping out of the house and only a handful wore it the proper way covering the nose and mouth. The Nigeria study that assessed the knowledge, attitude, and practices of the public around COVID-19 also reported a very low level of compliance with wear mask in the public space [20]. On the contrary, there was good compliance with wearing a mask in public places in Saudi Arabia [28].

In our study, the positive predictors of compliance to universal mask policy were age ≥ 40 years, and increase in level of education especially post-secondary education. This is interesting as these same factors were predictors of willingness to wear a face mask. This could be tied to the perceived risks which are higher, especially in the older age group and often a higher level of education translates to understanding of health information and consequently, better health behaviours. Our findings were similar to that reported in the community study which also found that the older age group, males and those with post-graduate education reported high compliance with the use of face masks compared to other category groups [28].

This study was not without limitations. The assessment was self-reporting and interviewer-administered with the potential for recall and social desirability bias.

## Conclusion

This study showed that there was a widespread willingness to wear a face mask in public places. Age and level of education were predictors of both willingness to wear mask and compliance to universal mask policy. Being older was found to be a significant predictor of willingness to wear mask and compliance to universal mask policy while having tertiary level of education was a strong predictor of the willingness to wear mask and compliance to universal mask policy. A major barrier to masking were that it causes discomfort and inconveniencing to wear.

Compliance with public health preventive measures remains a very important strategy to control any epidemic and the COVID-19 pandemic. This study was conducted early in the pandemic in Nigeria and many of the findings may have changed due to the prolonged pandemic. It remains constant that willingness does not entirely translate to actual compliance, our study suggests that willingness to wear a face mask influences compliance. Additional research is needed to ascertain evidence-based effective risk communication strategies to reach diverse groups especially those that are vulnerable and less likely to practice the wearing of masks. Repeated studies are needed to assess the compliance with public health preventive measures. The results of such studies could help public health practitioners and researchers in designing interventions for promoting behavioural change to reduce COVID-19 related morbidity and mortality and improve public health efforts.

## Data Availability

All data generated and analysed during this study are included in this published article.
